# Coordination Nanosheet-Based Electrochromic Supercapacitor with High Energy Storage, Switching Durability, and Long Optical Memory Properties

**DOI:** 10.1021/acsami.5c13795

**Published:** 2025-10-29

**Authors:** Susmita Roy, Sayan Halder, Sarda Sharma, Karumbaiah N. Chappanda, Chanchal Chakraborty, Masayoshi Higuchi

**Affiliations:** † Department of Chemistry, 209298 Birla Institute of Technology & Science (BITS) Pilani, Hyderabad Campus, Jawaharnagar, Samirpet, Hyderabad, Telangana 500078, India; ‡ Electronic Functional Macromolecules Group, 52747 National Institute for Materials Science (NIMS), 1-1 Namiki, Tsukuba 305-0044 ,Japan; § Department of Electronics and Communication Engineering, 563960 Amrita School of Engineering, Bengaluru, Amrita Vishwa Vidyapeetham, Bengaluru 560035, India; ∥ Sensors and Nano Electronics (SANE) Lab, School of Applied Engineering and Technology, 2254 Southern Illinois University Carbondale, Carbondale, Illinois 62901, United States; ⊥ Materials Center for Sustainable Energy & Environment (McSEE), Birla Institute of Technology and Science, Hyderabad Campus, Hyderabad 500078, India

**Keywords:** metallo-supramolecular polymers, coordination nanosheets, electrochromic supercapacitor, optical memory, redox-complementary counter material

## Abstract

Electrochromic (EC) supercapacitors have attracted considerable attention as energy storage systems integrated with optical functions. EC supercapacitors with high-performance and long-term optical memory properties were successfully fabricated by a combination of coordination nanosheets (CONASH), composed of Fe­(II) ions and a tristerpyridine ligand having a nonconjugated linker, and nickel hexacyanoferrate (NiHCF) as a redox-complementary counter material. The EC supercapacitor exhibited EC changes between purple and pale yellow with large optical contrast (57.4% at 556 nm), short switching times (1.28/1.69 s), exceptionally high coloration efficiency (619 cm^2^ C^–^^1^), significantly small energy consumption (3.6 mJ/cm^2^), and excellent EC switching stability of more than 50,000 cycles. The EC supercapacitor also demonstrated high volumetric capacitance (248.1 F/cm^3^), energy density (29.37 mW h/cm^3^), and power density (7.5 W/cm^3^), maintaining stable performance over 40,000 galvanostatic charge–discharge cycles. Most notably, the device showed a drastically reduced self-discharge property as only 33% optical contrast was returned after 36 h under open-circuit conditions, paving the way for an efficient energy storage solution by exploiting the long optical memory of the device. Combining superior EC functionality with robust supercapacitive performance, this study offers a foundation for sustainable energy technology.

## Graphical abstract

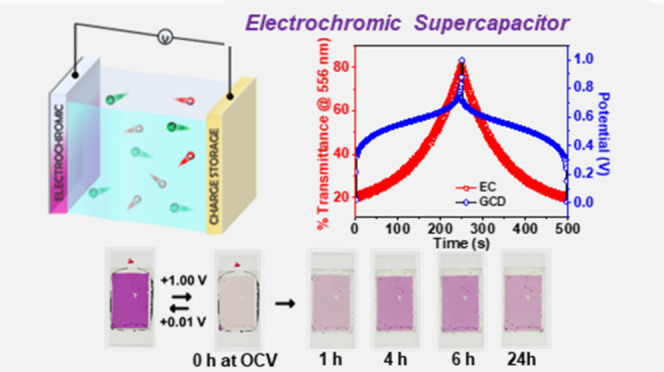

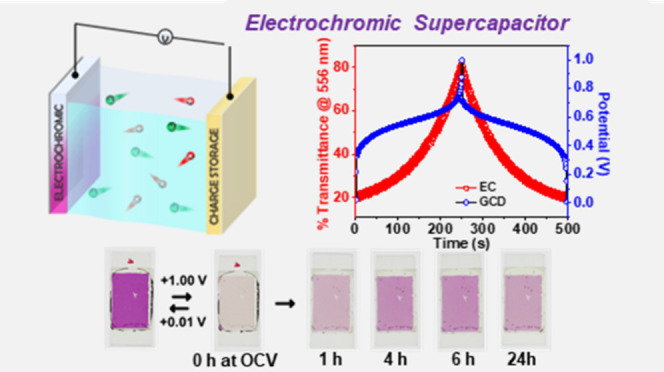

## Introduction

As innovative and exceptional energy storage devices, supercapacitors provide excellent power density, quick charge–discharge abilities, and excellent cycling stability, making them highly promising for various applications.^1^ However, with the advent of intelligent electronic goods, multifunctional supercapacitors, including flexible, wearable, self-healing, piezoelectric, etc., are increasingly needed.^2,3^ In this regard, electrochromic supercapacitors (ECSCs) have gained significant attention due to their ability to simultaneously change energy states with color, allowing visual determination of the energy state.^4,5^ Integrating energy storage and electrochromic (EC) functionality into a single device is feasible because they share similar mechanisms and device structures.^6,7^ An essential component of this integration is the kind of material used for the electrodes, the substantial voltage window, and the contrast in color between the charged–discharged states.^8,9^ Electrode materials should possess both EC and supercapacitive properties, such as PEDOT, PANI, PPy, NiO, WO_3_, TiO_2_, and V_2_O_5_, etc.^10–15^ However, challenges remain in making EC devices durable in the narrow electrochemical window of electrolytes (0–1.2 V for aqueous and 0–1.8 V for organic) compared to the driving voltage of EC devices, especially in solid-state EC devices.^16–21^ High driving voltage can decompose the electrolyte solvent, form gaseous byproducts, increase the internal resistance, and reduce cycling stability.^22^ High EC operating voltage also increases energy consumption, especially in large-scale or multiarray systems.^23,24^ To develop low-threshold voltage EC devices, strategies include enhancing the electric conductivity of EC materials, increasing the ionic conductivity of the electrolyte, judiciously designing the chemical structure for porous and nanoarchitecture morphology, and incorporating suitable counter charge-storage electrode layers.^25–27^ Despite these strategies, achieving ultralow threshold voltage and good energy saving and storage capabilities simultaneously remains challenging, necessitating new materials or mechanisms. Moreover, an EC device with high EC memory is energy-efficient because it does not need continuous power to sustain its new state after switching.^28,29^ To maximize the memory effect in EC devices, the open-circuit operation must minimize self- or residual or ambient oxidation and reduction. In the oxidative coloring of EC materials, the memory effect is related to the slower discharging of the stored energy. On the contrary, EC devices with rapid color switching times mainly depend on the swift ion during electrochemical processes and are operated through faster redox transition.^30–32^ Thus, the faster switching time is related to faster charging, especially in oxidative EC materials. So, the EC materials with high optical memory and faster switching times are desirable for providing EC devices with faster charging and slower discharging time, ideally to provide a Coulombic efficiency of around 100%. However, creating EC materials that combine high memory retention and fast switching speeds is challenging.

Metallo-supramolecular polymer (MSP) architectures have broadened the field of polymer and material science with applications in catalysis, display technology, molecular conductivity, and biomedicine.^33–35^ The oxidation state of the metal ion, and consequently the band structure, can be altered by applying an electrochemical potential, enabling diverse optical and spectro-electrochemical applications.^31,36^ MSPs and metal–organic frameworks (MOFs) are both coordination polymers, but they differ in their structural dimensionality, crystallinity, porosity, and nature of their charge-transport pathways. MOFs are highly ordered porous structures with permanent porosity and high crystallinity, while MSPs have amorphous structures, mechanical flexibility, and uniform film processability, which are advantageous for thin-film technology. Generally, the electrochromism of MSPs is driven by metal-to-ligand charge transfer (MLCT), and redox switching of the metal center alters the MLCT band, resulting in fast and reversible optical modulation. On the other hand, the EC activity of MOFs is usually derived from redox-active linkers or metal nodes.^37^

The 2D coordination nanosheets (CONASHs) are a special class of MSP that can be synthesized by coordination between organic ligands and metal cations through bottom-up interfacial complexation.^38,39^ Depending on the choice of metal ions and conjugation in ligands, the electrical conductivity of CONASHs can be tuned for high conductivity (10^–^^3^–10^3^ S/cm) due to π–d conjugation with a conjugated organic ligand or very low conductivity (<10^–^^10^ S/cm) utilizing the lack of planarity in the nonconjugated ligand.^38,40^ Exploiting the electronic and redox behaviors, CONASHs are used in photoactivity, electrocatalysis, trans-metalation, electrochromism, etc.^39,41,42^ In recent times, a few Fe­(II) or Co­(II) containing CONASHs have been reported as excellent EC materials for electrochromism, focusing on the color fastness by utilizing the advantages of the nanosheet structure and conjugated ligand environment in CONASHs.^39,41–48^ To converge the high optical memory with faster switching time, we have introduced nonconjugated ligands in Fe­(II)- or Co­(II)-based CONASHs.^29,49^

Conventionally, the redox mechanism of EC devices relies on the movement of a single type of cation or anion during the coloring or bleaching process. This is akin to the “rocking chair” model in energy storage devices.^50^ The mechanistic similarity of EC and battery-type energy storage made MSPs popular as indicative electrochromic energy storage devices (ECESD), as they could direct the energy status by their real-time color hue.^19,28,32,51–55^ However, CONASHs are not explored as much as ECESD despite their structural and dimensional superiority in the MSP category.^48^ On a few occasions, Higuchi et al. reported Fe­(II)- and phenanthroline-based CONASH for ECESD with a red-to-colorless EC transition and high capacitive behavior.^45,55^ In another study, Cong et al. reported a dual-redox-active CONASH incorporating a triphenylamine and phenanthroline–Fe­(II) complex where dual-redox centers provided mutual Faradaic contributions, synergistically enhancing both EC and pseudocapacitive energy storage performances.^56^ Additionally, Zhuang et al. demonstrated ultrathin CONASH for applying miniaturized flexible microsupercapacitors.^57^ Interestingly, all reported ECESD applications focused on fully conjugated ligand-based CONASH. However, the effect of nonconjugation within CONASH remains unexplored to date. Yet, nonconjugation in ligands can slow down the electron movement and restrict the electron hopping both in-plane and interplane, reducing the self-reduction tendency of the metal centers to improve the discharge times in availing the highest Coulombic efficiency during energy storage applications. Therefore, it is crucial to study the effect of nonconjugation on the ECESD performance in CONASH.

In our present work, we have fabricated an ECESD by assembling a nonconjugated ligand-based CONASH (Fe-3TPY) as anode and nickel hexacyanoferrate (NiHCF) as redox-complementary cathode to demonstrate the synergistic effects of Li^+^ ion insertion/extraction at the cathode and ClO_4_^–^ doping/dedoping at the anode while using LiClO_4_ as electrolyte. The nonconjugated linker decouples electronic communication between metal centers, suppressing electron transport and self-discharge while improving optical memory retention. It also minimizes π–π stacking, enabling flexible, low crystallinity, and crack-free nanosheets formation. Thus, it strategically enhances the redox site localization, film quality, electrochemical stability, and memory performance. Again, redox-active transparent complementary electrode-based EC dual-ion capacitors have garnered significant attention as they rely on the participation of both cations and anions from the electrolyte between the two electrodes, resulting in excellent EC performance.^28^ A key advantage is that the complementary electrode can reduce the overall operating voltage window while enhancing the devices’ coloration efficiency, specific capacity, and stability. The fabricated ECESD showcased notable EC performance in low-voltage window to change the color from pristine pink to pale yellow during EC behavior with a high optical contrast (Δ*T*) of 57.4% at 556 nm and fast EC switching times of 1.28 s for bleaching and 1.69 s for coloration, high coloration efficiency, and ultrahigh EC stability over 50,000 EC cycles. Utilizing the nonconjugated linkers in Fe-3TPY, the ECESD showed very high optical memory, retaining 33.3% of its optical contrast even after 36 h. The dual-ion-based ECESD also exhibited a high volumetric capacitance of 248.1 F/cm^3^ at 1 A/cm^3^ with a slight IR drop of 0.15 V. It demonstrated high energy and power densities of 34.45 mW h/cm^3^ and 7.5 W/cm^3^, respectively, with 88% retention of the initial capacitance after 40,000 GCD cycles. Notably, volumetric capacitance is more relevant to evaluate a compact and thin-film device’s energy storage performance. Since the active material mass in nanoscale-level films or coatings is extremely small, areal capacitance may not adequately reflect device performance. In contrast, changes in the film thickness at a given surface area may significantly affect the volumetric capacitance.^58^ In general, attaining both the EC and energy storage properties in a single system is challenging. This is because superior EC properties, such as higher coloration efficiency and faster switching time, tend to be realized with low charge densities, whereas better energy storage requires higher charge densities.^59,60^ To overcome this contradiction, we introduced the CONASH architecture to achieve superior EC properties at high charge densities. This architecture provides a large electroactive surface area for improved electric double layer capacitance and a short ion diffusion path, enabling both charge storage and rapid EC switching to coexist in ECESDs. Furthermore, the use of NiHCF as a thin, redox-active complementary counter electrode (CE) can improve charge-storage and supercapacitive properties. Furthermore, the ECESD demonstrated low energy consumption during electrochromism, even lower than that of conventional display technologies (LCD, OLED, etc.). The ECESD design and corresponding ameliorated properties of Fe-3TPY in this report offer a fresh notion and direction for creating a high-performance indicative ECESD.

## Experimental Section

### Materials and Instrumentation

All reagents in this study were reagent grade and were employed without further purification. The reaction solvents were extrapure dichloromethane (DCM) and ethanol (EtOH). Spectrochemically pure acetonitrile (ACN) was employed for cyclic voltammetry (CV), device fabrication, and spectroscopic analyses. S D Fine-Chem Limited supplied reagent grade ACN, DCM, EtOH, and acetic acid (AA). Specifically, 4′,4‴′-(1,4-phenylene)-bis­(2,2′:6′,2″-terpyridine), iron­(II)-acetate, 4′-chloro-2′,2:6′,2″-terpyridine, LiClO_4_, and Fe­(BF_4_)_2_·6H_2_O were acquired from Sigma-Aldrich. Tetra-*n*-butylammonium perchlorate (TBAP), propylene carbonate (PC), and poly­(methyl methacrylate) (PMMA) were sourced from TCI Chemicals (India) Pvt. Ltd. Furthermore, 2-(hydroxymethyl)-2-methylpropane-1,3-diol and powdered KOH were obtained from Sisco Research Laboratories Pvt. Ltd. (SRL)-India. ITO-coated glass slides with a resistivity of approximately 20 Ω and transmittance exceeding 90% were procured from Shilpa Enterprise, India. Millipore Milli-Q water with a resistivity of 18 MΩ cm was employed as needed. Water-based nickel hexacyanoferrate (w-NiHCF) was obtained from Cica-Reagent from Kanto-chemical Co., Inc. and used further for film preparation after dilution with DI water. Synthesis of (4′,4‴′-((2-(((2,2′:6′,2″-terpyridin)-4′-yloxy)-methyl)-2-methylpropane-1,3-diyl)­bis­(oxy))­di-2,2′:6′,2″-terpyridine) (3TPY), as shown in Scheme S1a in Supporting Information (SI), was conducted following our previously reported procedure.^29^ The details of the synthesis of Fe-3TPY film grown through interfacial bilayer polymerization using 3TPY ligand in DCM solution and Fe­(BF_4_)_2_·6H_2_O in water and the collection procedure of the film in ITO are provided in our previously reported procedure.^29^ A schematic of the synthesis and collection of the Fe-3TPY film is mentioned in Scheme S1b in the Supporting Information.

Electrochemical measurements were performed by using an ALS/CHI electrochemical workstation (Model 612B, CH Instruments, Inc.) with a two-electrode system. An integrated Ocean Optics modular spectrometer was connected to the electrochemical analyzer for monitoring the optical spectral behavior of the EC device upon potential application. A FEI, Apreo SEM instrument with a 30 kV operating voltage was used for field emission scanning electron microscopy (FESEM) to determine the film morphology. The film surface was gold-coated by sputtering with gold for 30 s to reduce the surface potential. The X-ray photoelectron spectral (XPS) study was conducted using a Thermo Scientific Multilab 2000 with AlKα radiation (1486.6 eV) operated at 15 kV and 10 mA (150 W). All the binding energies are with reference to C 1s at 284.85 eV. To check the powder X-ray diffraction (PXRD) pattern, CONASH’s flakes from EtOH were dropped on a Kapton holder, dried at 55 °C overnight, and subjected to the Rigaku MiniFlex XRD instrument.

### Fabrication of the Hybrid ECESD

In our present study, Fe-3TPY films were directly deposited on ITO glass substrates. Cracked or uneven regions were carefully removed to obtain a uniform film area. However, the NiHCF-coated electrode was obtained by spin-coating an aqueous-NiHCF solution at 1200 rpm on the conductive side of ITOs. NiHCF-coated ITOs were oven-dried at 70 °C overnight, whereas the Fe-3TPY-coated films were air-dried first and then oven-dried at 70 °C for 2 h. The quasi-solid Li-ion-based gel-electrolyte was prepared with 0.9 g LiClO_4_ in 21 mL ACN and 6 mL PC, followed by adding 2.1 g PMMA. The mixture was stirred at room temperature for 8 h to become a transparent viscous state. The point to be mentioned is that the gel-electrolyte bottle was tightly sealed after each use and stored in a desiccator with low humidity. Finally, the dried film-coated ITOs were sandwiched with the gel electrolyte and kept at room temperature for drying before use.

## Results and Discussion

### Synthesis and General Characterizations

The flexible nonconjugated ligand 3TPY was synthesized according to our previously reported protocol.^29^ This included the reaction between 2-(hydroxymethyl)-2-methylpropane-1,3-diol and 4′-chloro-2,2′:6′,2″-terpyridine in the KOH base in the presence of anhydrous DMSO, as represented in Scheme S1a (SI). The synthesized 3TPY was thoroughly characterized by NMR and mass spectroscopy, with detailed results provided in our previous literature.^29,49^ Furthermore, as illustrated in Scheme S1b, the synthesis of Fe-3TPY nanosheets was carried out under static conditions at the interface of 15 mL DCM containing 0.1 mM of 3TPY and 15 mL water containing 50 mM of Fe­(BF_4_)_2_·6H_2_O. The formation mechanism of the Fe-3TPY film relies on the complexation between the TPY units of adjacent ligands, facilitated by the introduction of suitable Fe^2+^ ions diffusing from the aqueous subphase.^38,61^ As previously discussed, to control the film’s thickness and color intensity, the reaction parameters, such as the concentration of metal and the duration of layering in the reaction setup, can be adjusted.^46^ Although other in situ polymerization methods, such as electrochemical deposition, exist, only interfacial polymerization allows the formation of two-dimensional CONASHs. DCM and water were chosen as the solvent because they are immiscible. The organic ligand is soluble only in DCM, and the Fe­(II) salt is soluble only in water. Moreover, our previous report determined the stoichiometric complexation ratio between the 3TPY ligand and Fe^2^^+^ ions is to be 2:3 by using UV–vis titration by gradually adding Fe^2^^+^ ions to a solution containing 3TPY.^29^ The chemical structure of Fe-3TPY is represented in Figure 1a by considering the above-mentioned stoichiometric ratio. The comparative UV–Vis spectroscopy analysis (Figure 1b) of 3TPY and Fe-3TPY revealed a notable spectral shift of the peaks. Initially, for 3TPY, a distinct absorption peak at 272 nm was observed, attributed to the π–π* transition within its aromatic unit. Upon complexation with Fe^2+^, this transition underwent a significant red shift to 312 nm in Fe-3TPY. Additionally, a minor peak appeared at 360 nm, corresponding to the metal (Fe^2+^) d–d transition, along with an intense peak at 556 nm owing to the MLCT (d of Fe^2+^ to π* of 3TPY) transition, also responsible for the intense purple color of the CONASH. The FESEM image consistently revealed a homogeneously smooth, flat nanosheet structure throughout its surface (Figure 1c). The average film thickness of the Fe-3TPY film was maintained at 350 ± 10 nm through AFM imaging, as shown in Figure 1d, by preparing an interfacial nanosheet over 48 h.^29^ The bonding environment and structural connectivity of Fe-3TPY were comprehensively examined through XPS analysis. In the core-level scan of N 1s, as shown in Figure 1e, a distinct peak corresponding to the nitrogen atoms of 3TPY was observed at 398.17 eV, which exhibited a slight shift to 399.92 eV in Fe-3TPY.^62^ Analysis of the Fe 2p core-level scan of Fe-3TPY (Figure 1f) revealed two distinct peaks at Fe 2p_3/2_ (708.66 eV) and Fe 2p_1/2_ (721.23 eV). This outcome explicitly confirmed the coordination of terpyridine moieties with Fe­(II) within the Fe-3TPY nanosheets. Additionally, the XPS analysis-based atomic ratios indicated N/Fe ratios of 6.63:1.17, which were very close to the theoretical N/Fe ratios of the ideal standard bis­(terpyridine)–Fe­(II) complex of 6:1. The Raman spectra (Figure 1g) of CONASHs exhibited a downward shift in the characteristic aromatic C=C or C=N stretching vibrations compared to the free ligand, confirming the successful coordination of Fe­(II) to 3TPY. Additionally, the PXRD pattern (Figure 1h) of CONASHs showed an amorphous nature with a broad peak centered at 2θ = ∼21.9, corresponding to a short-range interaction at 4.05 Å. The extensive optical, structural, and thermal characterizations of Fe-3TPY are provided in our earlier report.^29^

**Figure 1:**
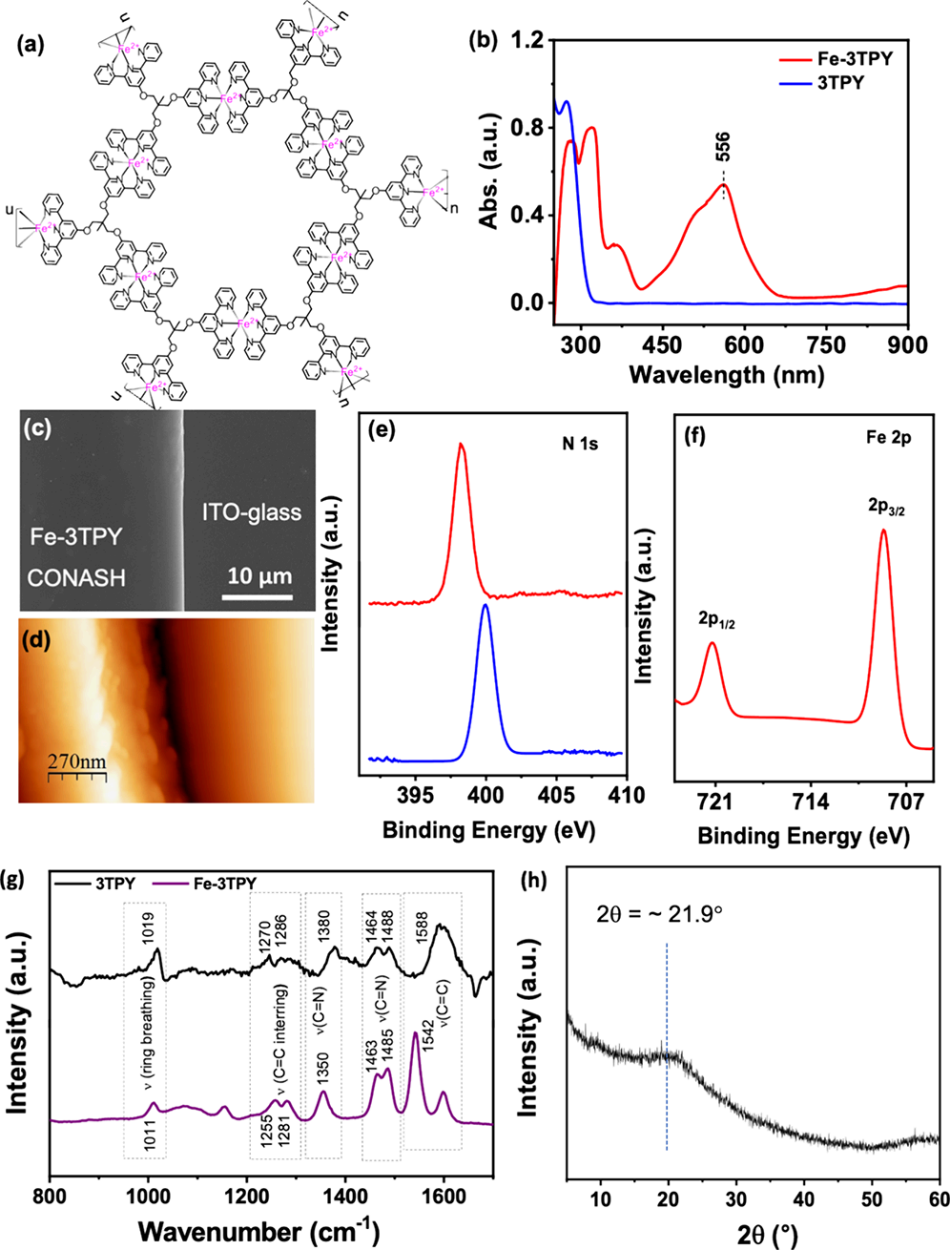
(a) Chemical structure of Fe-3TPY. (b) UV–vis spectra. (c) FESEM images of Fe-3TPY. (d) AFM image of the CONASH film. Core-level XPS spectra of (e) N 1s for 3TPY (blue) and Fe-3TPY (red) and (f) Fe 2p for Fe-3TPY. (g) Raman spectra of CONASH film (violet line) and the ligand 3TPY (black) on a glass slide. (h) PXRD pattern of collected CONASH flakes on a Kapton holder.

### Electrochemical and EC Properties of ECESD

The Fe-3TPY and NiHCF-based sandwich-shaped gel-based quasi-solid-state ECESD was made utilizing a Fe-3TPY-coated ITO as the active working electrode (WE) material and NiHCF-coated ITO as the CE with a LiClO_4_ and PMMA-based semigel electrolyte in between electrodes. Here, the thickness of the Fe-3TPY layer was 350 ± 10 nm, as mentioned, and the average thickness of the NiHCF layer was maintained at 140 ± 10 nm (given in the inset of Figure 2a). Before quantitatively analyzing the energy storage properties, the energy storage behavior of the Fe-3TPY-based ECESD was assessed through a CV study by varying scan rates. At the scan rates between 25 and 200 mV s^–1^, the ECESD exhibited a distinct reversible redox peak identified with the Fe^2^^+^/Fe^3^^+^ redox couple (Figure 2a), indicating the characteristic reversible faradic behavior of ECESD. Notably, increasing scan speeds led to higher oxidative and reductive current densities, indicating the ECESD’s low internal resistance and rapid charge-transfer kinetics, with the charge storage mechanism described by eq 1:

$$i=av^{b}$$ 1

where *a* and *b* are configurable parameters, *v* is the scan rate, and *i* is the peak current. We determined *b*-values of 0.680 for anodic and 0.708 for cathodic current maxima by plotting log­(*i*) vs log­(*v*) (Figure 2b). This demonstrated the predominant pseudocapacitive behavior of the Fe-3TPY-based ECESD and facilitated quantitative evaluation of diffusive and capacitive contributions to energy storage by using eq 2:

$$i=k_{1}v+k_{2}v^{1/2}$$ 2

where *k*_1_ and *k*_2_ are constants derived from the *i*/*v* ^1/2^ versus the square root of the scan-rate plot.

**Figure 2:**
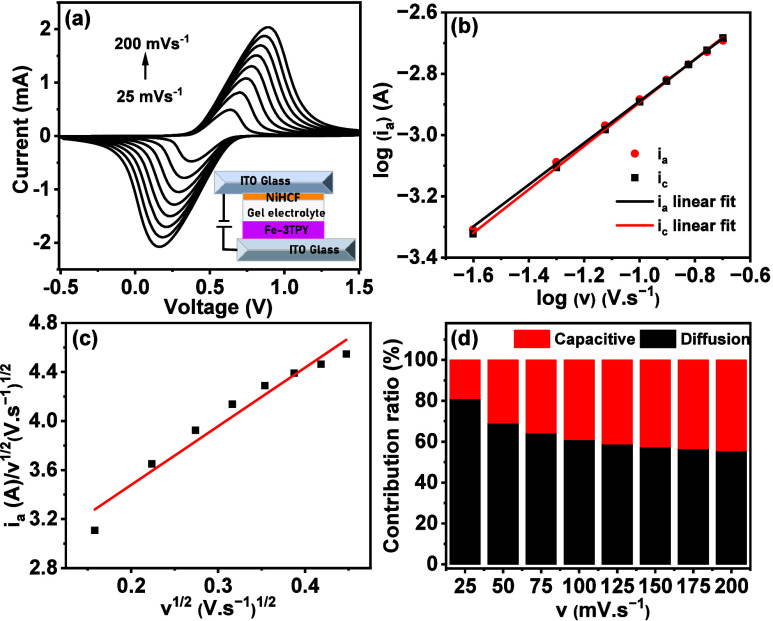
(a) Scan-rate (ν)-dependent CV study of Fe-3TPY- and NiHCF-based ECESD. (b) Plot for calculating the b value from the anodic (*i*_a_) and cathodic (*i*_b_) peaks for the ECESD. (c) *i*/ν^1/2^ versus the square root of the scan-rate plot to determine the *k*_1_ and *k*_2_. (d) Pseudocapacitive and diffusive contributions of Fe-3TPY- and NiHCF-based ECESD at different scan rates.

From Figure 2c, we determined *k*_1_ as 0.00556 and *k*_2_ as 0.00223. Consequently, we calculated the pseudocapacitive contribution of the Fe-3TPY/NiHCF ECESD to be over 80% at a scan rate of 25 mV s^–1^. The pseudocapacitive contribution of EECSD decreased with increasing scan rates, reaching 55% at a 200 mV s^–1^ scan rate (Figure 2d). These results demonstrate Fe-3TPY’s potential for high-power energy storage applications, making it a viable option for supercapacitor technology.

To gain insight into the electrochromism behavior of asymmetric ECESDs, we monitored the transmittance spectrum change of the ECESD when applying different voltages. It is worth noting that the transmittance was measured by subtracting the background of the ITO/glass substrate with an electrolyte gel. The transmittance spectra of ECESD revealed the disappearance of the MLCT peak at 556 nm (Figure 3a), with a reversible color change from pristine pink to a pale yellowish of the ECESD, as shown in Figure 3b. The CV plot revealed that ECESD exhibited an oxidation peak for Fe^2^^+^ to Fe^3^^+^ at +0.64 V and a corresponding reduction peak for Fe^3^^+^ to Fe^2^^+^ at +0.37 V, observed at a scan rate of 25 mV/s (Figure 2a). The color change of the ECESD is linked to the reversible Fe^2^^+^/Fe^3^^+^ redox transition, resulting in the complete disappearance of the 556 nm MLCT peak within a potential window of 0.01 to 1.0 V. The Fe-3TPY-based ECESD exhibited a distinct color change, resulting in a high optical contrast (Δ*T*) of 57.4% at 556 nm. It is worth noting that the incorporation of NiHCF as a CE material did not hamper the overall transmittance of the device, as the NiHCF electrode revealed a transparent pale yellowish color (Figure S1 in Supporting Information) originating from the d–d transition in the material.

**Figure 3:**
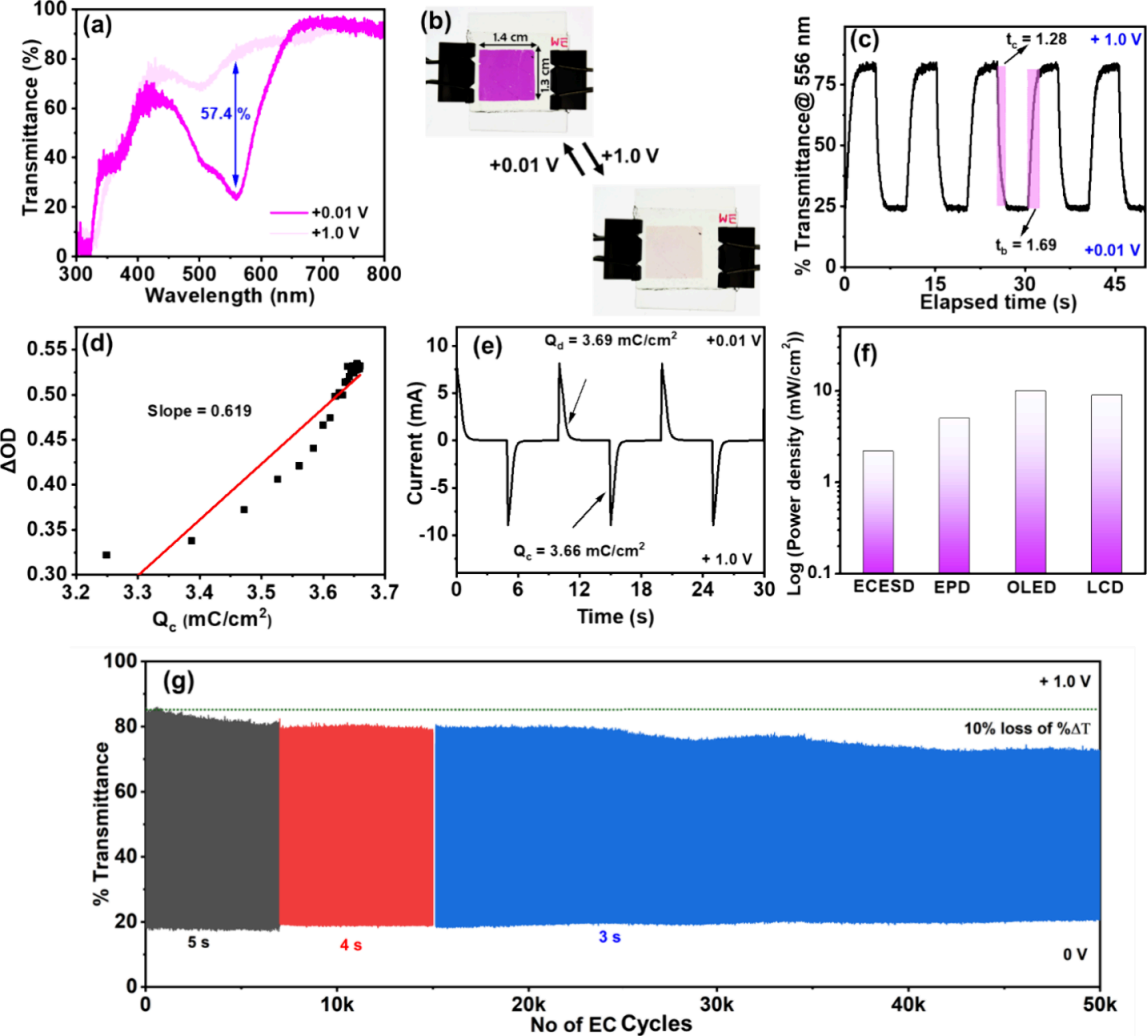
(a) Transmittance spectral change of the ECESD with an operating voltage of +1.0 and +0.01 V. (b) Photograph of the color change for the fabricated ECESD (film size: 1.4 × 1.3 cm^2^) at different voltages. (c) EC response time determination of the ECESD is based on a 95% change in transmittance. (d) Calculation of coloration efficiency for the Fe-3TPY-based ECESD. (e) Chronoamperometry charge–discharge plot during the electrochromism of the ECESD. (f) Power densities of several display technologies: a comparison of the OLED (organic light-emitting diodes, active matrix),^63^ LCD (liquid crystal display, active matrix, LTPS TFT),^64^ EPD (electrophoretic displays, active matrix),^65^ and the fabricated ECESD in the context of power consumption. (g) EC switching performance of Fe-3TPY-NiHCF-based ECESD by applying +1.0 and 0.01 V with 5, 4, and 3 s interval time at each voltage, monitoring the transmittance change at 556 nm.

The fundamental EC parameters, such as the switching speed, coloration efficiency, and device durability, were determined later for the practical applicability of EC materials. The EC parameters of Fe-3TPY were determined using double potential chronoamperometric studies, sweeping the potential between +1 and 0.01 V. The switching times, defined as the time taken for a 95% change in Δ*T*, were calculated from the transmittance vs time plot shown in Figure 3c. In general, ECDs with a fast-to-moderate switching time and high coloration efficiency (η) are desirable for commercial applications. In this study, the switching times of ECESD were determined to be 1.28 s for coloration (*t*_c_) and 1.69 s for bleaching (*t*_b_). “η” is a measure of the device’s energy efficiency, which can be calculated by dividing the quantity of charge injected or ejected (*Q*_d_) per unit area by the ratio of optical density change (ΔOD). The “η” of the CONASH-based hybrid ECESD, calculated from the slope of the ΔOD vs *Q*_d_ plot in Figure 3d, was 619 cm^2^ C^–^^1^, which is higher than that of previously reported Fe­(II)-containing MSPs.^29,47,53,54^ We also derived the power and energy consumption of the ECESD from the chronoamperometric charge–discharge plot given in Figure 3e. The power and energy consumed by the CONASH-based hybrid ECESD during electrochromism were 2.18 mW/cm^2^ and 3.6 mJ/cm^2^, respectively, calculated by using eq 3 as mentioned in the SI at an operating voltage of +1.0 and +0.01 V by taking the 1.4 × 1.3 cm^2^ film size. We also compare the energy consumption data of ECESD with the conventional display technologies in Figure 3f, revealing even lower energy consumption in our ECESD compared to other conventional display technologies.^63–65^ In the cycle stability study, we evaluated the long-term switching capacity of the ECESD within specified holding intervals of 5, 4, and 3 s, as illustrated in Figure 3g. As the ECESD showed faster switching and very low coloration and bleaching times, we anticipated swift and reversible switching in any holding times beyond 2 s. Remarkably, the ECESD exhibited exceptional cycle stability, enduring over 50,000 cycles of operation. By accurately analyzing the initial and final optical contrast (Δ*T*), we determined that the ECESD retained approximately 90% of its initial Δ*T* following 50,000 repeated switching events. This endurance provides durable performance of the ECESD equivalent to a remarkable 14-year lifespan based on a usage scenario of 10 cycles per day.^66,67^ This impressive durability is attributed to this device’s extremely low operational voltage (0.01–1 V) and reversible charge-balancing redox reactions occurring between the WE and CE. This highlights the robustness and suitability of fabricated ECESD for sustained practical applications in various fields.

High optical memory within an ECESD is an essential factor in assessing the power efficiency of smart windows. Typically, in an ECESD, the ability of a specific redox state, characterized by its color or bleaching level, to maintain its originality over an extended period in open-circuit conditions signifies the potential for creating a power-efficient device.^29,47,68^ In this work, after attaining the fully bleached state at +1 V bias, we continually monitored transmittance at 556 nm and the change in optical color to examine the EC memory of the Fe-3TPY-based ECESD. Herein, Figure 4a,b depicts the regeneration of transmittance and optical color over time under open-circuit conditions, starting from a fully bleached state. The observed gradual increment in transmittance peak and development of a pink hue over time resulted from the self-reduction of colorless Fe^3+^ centers by residual electrons from the electrode under open-circuit conditions.^29^ Interestingly, the ECESD demonstrated only a 33.3% recovery of its original color state after 36 h (Figure 4c). The electron transport activities between nearby Fe centers via the ligand π cloud and from the electrode to Fe^3+^ centers within the MSP in the bleached state play crucial roles in determining EC memory. In the Fe-3TPY system, the nonconjugated nature of the 3TPY ligand restricted electron hopping through the ligand’s π cloud, showcasing a highly extending EC memory of Fe-3TPY-NiHCF-based hybrid ECESD. It is noteworthy that the EC optical memory of our ITO/Fe-3TPY//NiHCF/ITO-based ECESD is not only one of the best in the MSPs or CONASH category^47^ but also enhanced a lot compared to our earlier report of ITO/Fe-3TPY/ITO-type devices,^29^ which showed 50% of coloration recovery only after 25 min in open-circuit conditions. Incorporating NiHCF as the redox-complementary CE in the devices can reduce the number of residual electrons during the oxidation of Fe-3TPY in the WE to increase the EC optical memory in the hybrid device.

**Figure 4:**
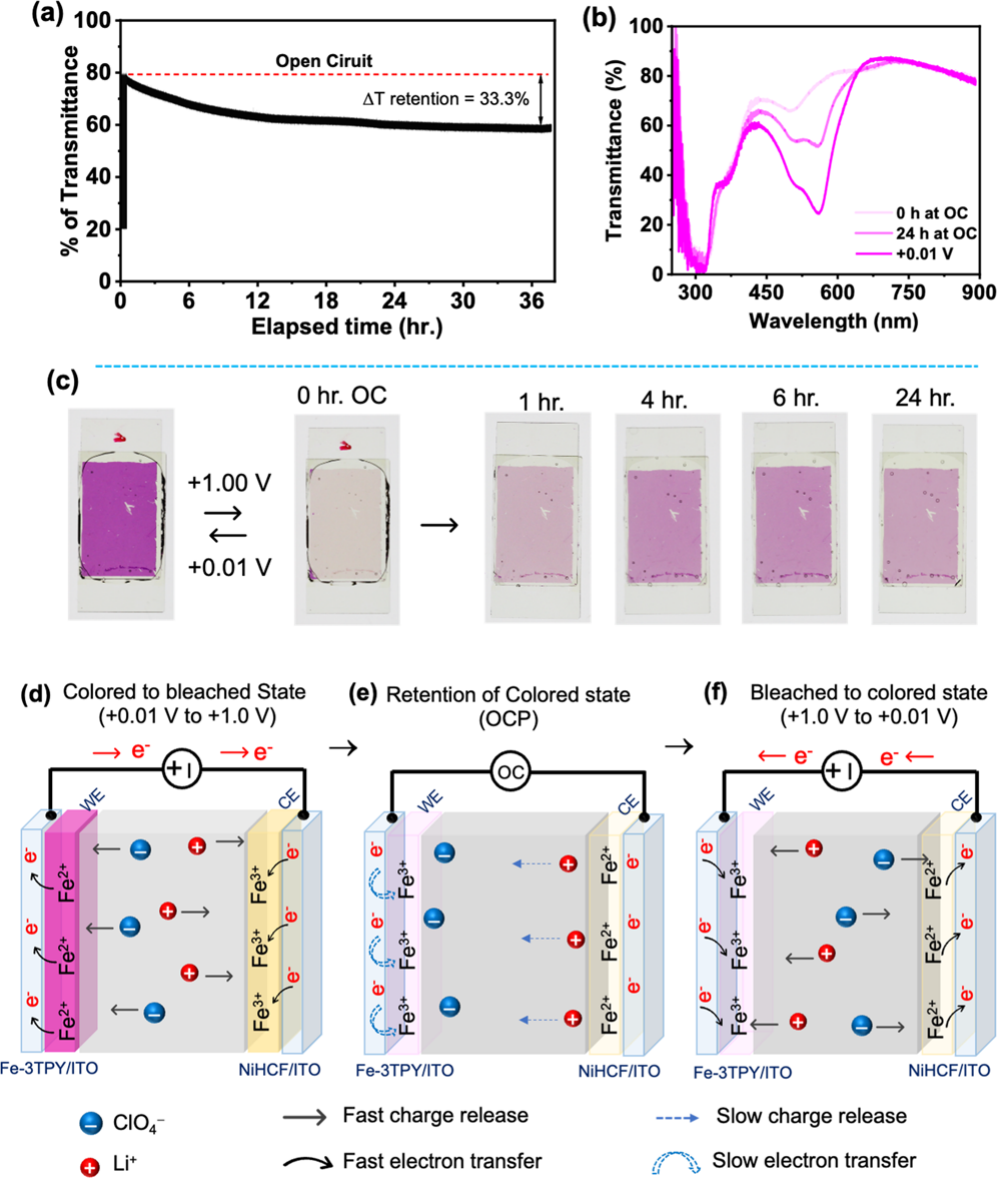
(a) Decrease in transmittance of the Fe-3TPY- and NiHCF-based ECESD (film size: 1.8 × 1.1 cm^2^) from the bleached state to the colored state at 556 nm under open-circuit conditions. (b) Complete transmittance spectrum of the ECESD at different time intervals. (c) Photographs of the ECESD under open-circuit conditions at various times after reaching the bleached state. A schematic representation for the mechanism of electrochromism of ECESD upon applying different voltages is shown: (d) dark mode to bright mode, (e) retention of bright/bleached mode, and (f) bright mode to dark mode.

Moreover, it is essential to understand the electrochemical reactions occurring between the WE and CE to comprehend the EC phenomenon of the ECESD. In the diagram given in Figure 4d, in the colored mode, the Fe-3TPY layer is distinguished by its pink hue, which indicates the presence of Fe^2+^ ions. Conversely, the NiHCF layer is represented in a light yellow shade, indicating the presence of Fe^3+^ ions. The electrolyte LiClO_4_ facilitates electrochemical processes between the two electrodes. We propose a mechanistic insight into the electrochemical redox reactions occurring within the device, defined by eqs 3–7.

Electrochemical reaction during bleaching of ECESD:

At the WE:

$$[{\mathrm{Fe}}^{2+}-3\mathrm{TPY}{]}_{n}.[{\mathrm{CH}}_{3}{\mathrm{COO}}^{-}{]}_{2n}+n{{\mathrm{ClO}}_{4}}^{-}\to [{\mathrm{Fe}}^{3+}-3\mathrm{TPY}{]}_{n}[{\mathrm{CH}}_{3}{\mathrm{COO}}^{-}{]}_{2n}[{{\mathrm{ClO}}_{4}}^{-}{]}_{n}+ne^{-}$$ 3

At CE:

$$n{{\mathrm{Ni}}_{1.5}}^{2+}[{\mathrm{Fe}}^{3+}(\mathrm{CN}{)}_{6}]+ne^{-}+n{\mathrm{Li}}^{+}\to n{\mathrm{Li}}^{+}{{\mathrm{Ni}}_{1.5}}^{2+}[{\mathrm{Fe}}^{2+}(\mathrm{CN}{)}_{6}]$$ 4

Electrochemical reaction during coloring of ECESD:

$$[{\mathrm{Fe}}^{3+}-3\mathrm{TPY}{]}_{n}[{\mathrm{CH}}_{3}{\mathrm{COO}}^{-}{]}_{2n}[{{\mathrm{ClO}}_{4}}^{-}{]}_{n}+ne^{-}\to [{\mathrm{Fe}}^{2+}-3\mathrm{TPY}{]}_{n}\cdot [{\mathrm{CH}}_{3}{\mathrm{COO}}^{-}{]}_{2n}+n{{\mathrm{ClO}}_{4}}^{-}$$ 5

$$n{\mathrm{Li}}^{+}{{\mathrm{Ni}}_{1.5}}^{2+}[{\mathrm{Fe}}^{2+}(\mathrm{CN}{)}_{6}]\to n{\mathrm{Li}}^{+}+n{{\mathrm{Ni}}_{1.5}}^{2+}[{\mathrm{Fe}}^{3+}(\mathrm{CN}{)}_{6}]+ne^{-}$$ 6

As a result, the predicted redox potential of the ECESD can be defined as

$$E_{\mathrm{voltage}\;\mathrm{window}}=E_{\mathrm{Ox},\mathrm{WE}}-E_{\mathrm{Red},\mathrm{CE}}$$ 7

When a positive voltage is applied, the oxidation reaction transpires on the Fe-3TPY film (resulting in the conversion of Fe^2+^ to Fe^3+^ at the WE), inducing a color transition from pristine pink to a pale yellowish hue (eq 3). Simultaneously, to maintain charge neutrality within the system, a reduction reaction takes place on the NiHCF film (involving the conversion of Fe^3+^ to Fe^2+^) at the CE (eq 4). In contrast, Li^+^ and ClO_4_^–^ ions migrate toward opposite electrodes (Figure 4d). Conversely, during the coloration phase, the reverse reactions occur at each electrode, with reduction transpiring at the WE and oxidation at the CE (eqs 5 and 6), as depicted in Figure 4f. In open-circuit conditions, after fully bleaching the ECESD, the generated Fe^3+^ ions undergo self-reduction by taking the residual electrons from electrodes (Figure 4e). The inherent nonconjugation ligand can slow down the electron hopping to reduce the self-reduction of Fe-3TPY. Again, the NiHCF component in CE is critical as a charge source/sink, facilitating the swift and facile redox reactions within the Fe-3TPY system. It can also consume the electrons for its reduction during bleaching, effectively reducing the number of residual electrons in WE in open-circuit conditions after bleaching to increase the optical memory. Additionally, the redox potential of the NiHCF (in Figure S1c in Supporting Information) regulated the overall potential of Fe-3TPY-NiHCF-based hybrid ECESD to obtain an overall potential window of +0.01 to +1.0 V.

### Capacitive Property of ECESD

The energy storage property of ITO/Fe-3TPY/LiClO_4_/NiHCF/ITO-based ECESD was examined by galvanostatic charge–discharge (GCD) and electrochemical impedance spectroscopy (EIS) techniques. As discussed earlier, the nonlinear CV curves of the ECESD showed excellent reversible redox behavior, which indicated that the Fe-3TPY-based ECESD disclosed an effective charge-storage pseudocapacitive system. The capacity to store charge in Fe-3TPY-based ECESD was examined by a GCD study at different current densities ranging from 1 to 15 A/cm^3^ in the +0.01 to +1.0 V potential window (Figure 5a). The nonlinear charge–discharge curves in the GCD study again confirmed the predominant pseudocapacitive charge-storage mechanism for the electrodes, which prevailed by surface faradic reaction at both electrodes. The ECESD showed a negligible *iR* drop of only 0.15 V in the GCD behavior. The minimal *iR* drop (Figure S2a in Supporting Information) originated from internal resistance caused by the electrolyte and electrode–electrolyte contact. Additionally, including a NiHCF layer as a charge-storage layer would probably increase facile ion diffusion, resulting in a minimal *iR* drop for this ECESD. The volumetric capacitance was calculated as 248.1 F/cm^3^ at 1 A/cm^3^ and slightly decreased to 211.5 F/cm^3^ at 15 A/cm^3^ (Figure 5b), indicating the ECESD’s rapid and high-rate charge-storage ability. To understand the charge-transport properties of the ECESD, we performed an EIS study in the 5 Hz to 1 kHz frequency range. The Nyquist plot (Figure 5c) revealed an interfacial charge-transfer resistance (*R*_CT_) of 180.2 Ω, and the slope in the lower frequency region showcased the ECESD’s diffusive behavior. After 40,000 consecutive charge–discharge cycles at a current density of 15 A/cm^3^, the constructed ECESD demonstrated ∼80% retention of the initial remarkable long-term charge–discharge stability, retaining approximately 80% of its initial volumetric capacitance (Figure 5d). Additionally, Figure S2b in Supporting Information illustrates this trend’s first and last GCD cycles. Furthermore, the Coulombic efficiency of the ECESD remained at 97% after completing 40,000 cycles, revealing its remarkable performance for commercial applications. At a current density of 1 A/cm^3^, the ECESD exhibited a power density of 0.5 W/cm^3^ and an energy density of 34.45 mW h/cm^3^. These values improved to 7.5 W/cm^3^ for power density and 29.37 mW h/cm^3^ for energy density at a higher current density of 15 A/cm^3^, as shown in Figure 5e. The intentional selection of a redox-active CE within the ECESD promoted redox activities between the anode and cathode, enabling a reversible push–pull effect that enhances the energy storage performance of Fe-3TPY- and NIHCF-based ECESDs. The volumetric energy and power densities of the hybrid ECESD were compared to those of previously published high-performance ECESD in a Ragone plot, as shown in Figure 5f. Uncracked smooth surface of the Fe-3TPY film after prolonged ECESD operation, confirmed by SEM, indicates the high durability of the nanosheets (Figure S3, Supporting Information). The above data demonstrate the efficient energy storage performance of the ECESD, highlighting the active participation of both electrode materials in the redox mechanism. This synergistic interaction enhances the overall performance, as each material contributes to the charge-storage and transfer processes. The Fe-3TPY and NiHCF electrodes work in tandem to provide high capacity and stability, ultimately producing robust and effective energy. We also fabricated a device using a larger film (3 × 2.4 cm^2^) by the interfacial polymerization method (Figure S4, Supporting Information). The ECESD using the larger film exhibited similar EC behavior at an applied voltage of +1.5/+0.01 V. The calculated energy consumption and power consumption of the device were 3.8 mJ/cm^2^ and 2.2 mW/cm^2^, respectively, which are consistent with the smaller ECESD described above. However, fabricating much larger films remains challenging due to the need for precise control of the interface. Further research is needed to design vibration-free platforms, develop optimized vessels, and achieve controlled solvent removal to enable mass production of large, crack-free films.

**Figure 5:**
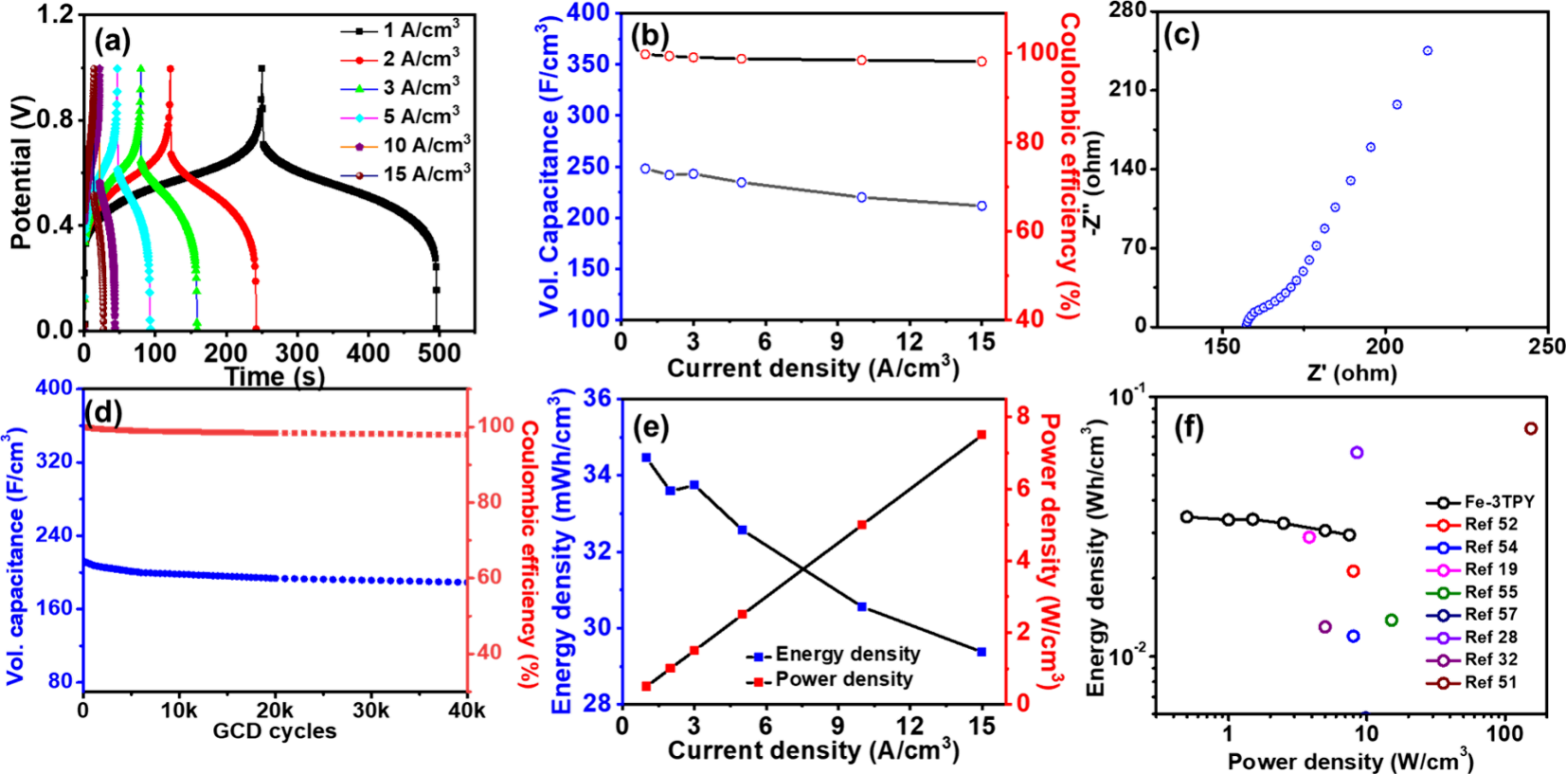
(a) GCD profiles of Fe-3TPY- and NiHCF-based ECESD at various current densities ranging from 1 to 15 A/cm^3^. (b) Volumetric capacitance change plotted against Coulombic efficiency at different current densities. (c) Nyquist plot of the ECESD. (d) Retention plot showing volumetric capacitance and Coulombic efficiency after 40,000 GCD cycles at 15 A/cm^3^. (e) Power and energy densities of the ECESD at various current densities. (f) Ragone plot of the ECESD.

Integrating high-performance energy storage and EC behavior is very important for the device to be used as an indicative supercapacitor for next-generation electronic devices. While measuring the energy storage performance, the ECESD changes its observable color depending on the device’s charge state, potential, and redox state. We monitored the in situ transmittance change of the hybrid ECESD at 556 nm during the charging and discharging process through GCD at a current density of 1 A/cm^3^ (Figure 6a). The figure provided the correlation between the transmittance and stored energy state of the ECESD, which allows users to predict the amount of charge storage by observing the color variation of the ECESD. The Fe-3TPY-based ECESD changes from pristine pink in its fully discharged state to a pale yellowish color when fully charged, as shown in Figure 6a inset. This demonstrates the correlation between charge-storage level and observable transmittance change. Furthermore, we have measured the transmittance changes of the devices during the charging–discharging in different current densities starting from 1 to 15 A/cm^3^. As shown in Figure 6b, the results revealed that our ECESD could track the color change over this wide range of current densities.

**Figure 6:**
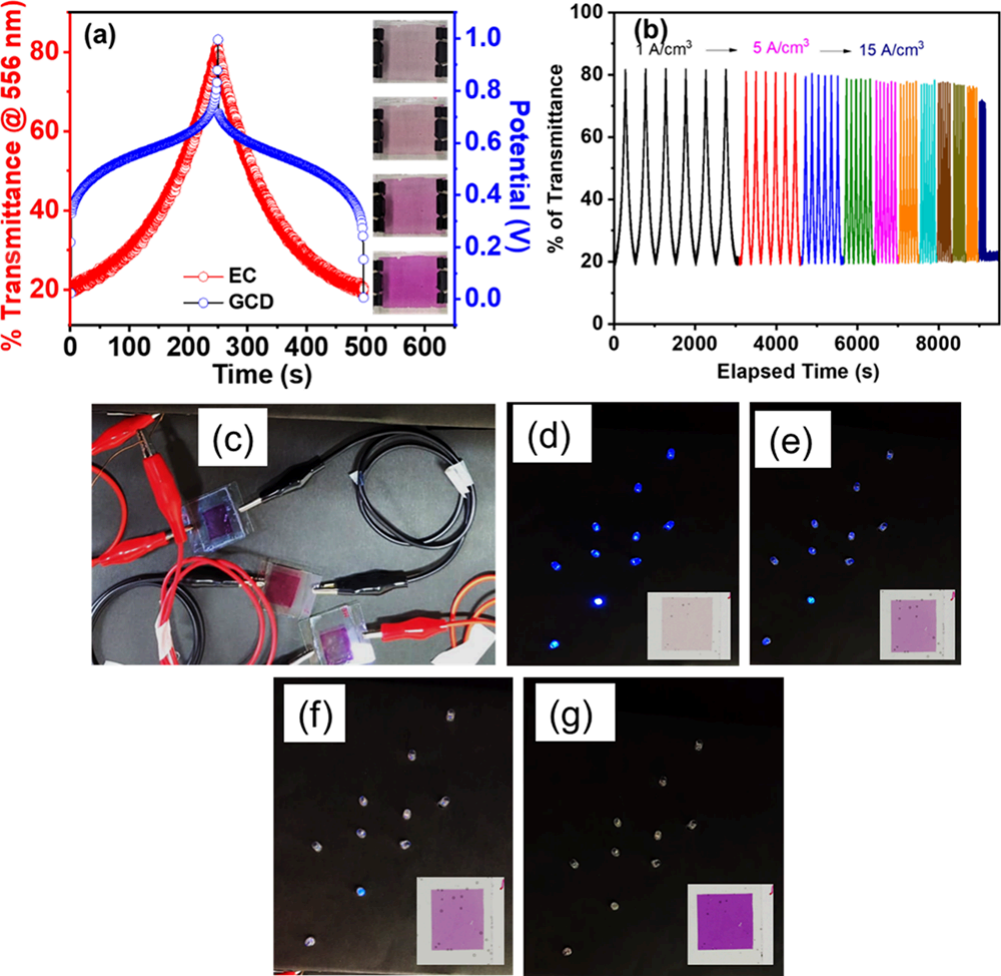
(a) Collective transmission change and GCD prolife at 1 A/cm^3^ current density with the corresponding color change of the ECESD at different voltage states in the inset. (b) Transmittance changes of the device when different current densities are applied, from 1 to 15 A/cm^3^. (c) Demonstration of three ECESDs in a series for light illumination of a 10-blue LED panel. (d–g) Gradual color generation of the ECESD from the fully charged bleached state, with the diminishing brightness of the LED panel over time.

The EC energy storage performance of the nonconjugated Fe-3TPY-based ECESD was compared with a conjugated ligand-based MSP Poly-Fe, maintaining a similar device architecture. A chemical structure and the synthesis protocol of Poly-Fe are mentioned in Figure S5a. In our previous article,^67^ we have shown the detailed durable EC performance of a poly-Fe-based EC device with NiHCF as a counter material. As shown in Figure S5b, the device showed a reversible purple to bleached EC transition in the operating voltage window of +1.2/+0.01 V with an optical contrast of 55% at 580 nm (Figure S5c in Supporting Information). The Poly-Fe- and NiHCF-based fabricated asymmetric device revealed comparatively much lower optical memory, as it can regain ∼17.8% of the initial optical contrast after 75 min, where Fe-3TPY-/NiHCF-based ECD could gain only ∼10% of color after 75 min, as shown in Figure 4a. The comparatively low optical memory in the Poly-Fe system is basically due to the conjugated configuration at the ligand that helped in faster electron transfer or electron hopping inside the system under open-circuit conditions to the self-reduction of the Fe­(III) center. The GCD study of the Poly-Fe-/NiHCF-based device revealed similar nonlinear behavior with a comparatively high *iR* drop of 0.5 V (Figure S5d in Supporting Information), due to a certain self-reduction tendency of the device in the presence of a fully conjugated system. The volumetric capacitance of the device was further calculated as 32 F/cm^3^ at 0.05 A/cm^3^, further decreasing to 23.5 F/cm^3^ at 1 A/cm^3^ (Figure S5e in Supporting Information). These observations suggested that the intentional incorporation of a nonconjugated system provided high optical memory and high volumetric capacitance with a slightly higher voltage window.

Moreover, we compared the EC energy storage performance of the Fe-3TPY-based device in the absence of NiHCF by fabricating the device as ITO/Fe-3TPY/LiClO_4_/ITO. Due to the absence of NiHCF, the operating voltage window of the device increased to +2.5 to −2 V, as shown in Figure S6a in Supporting Information. The GCD study of the device in Figure S6b revealed nonlinear behavior with a very high *iR* drop of approximately 2.5 V. This large *iR* drop resulted in decreased performance, with the volumetric capacitance calculated at only 32 F/cm^3^ at 0.05 A/cm^3^, further decreasing to 23.5 F/cm^3^ at 1 A/cm^3^ (Figure S6c in Supporting Information). The GCD cyclic performance in Figure S6d showed a significant capacitance loss within just 1000 cycles. The EC study also revealed that the optical contrast of the device slightly decreased to 53% (Figure S6e in Supporting Information). The optical memory of the device was drastically affected, as it regained its complete optical contrast within 60 min in open-circuit conditions (Figure S6f), indicating a fast charge loss of the energy storage device. These observations suggest that incorporating suitable CE materials can enhance the dual-ion-based redox mechanism. Each electrode’s push–pull effect improves energy storage and EC performance.

Moreover, we have compared the key performance parameters of the Fe-3TPY system with those of the previously reported MSP-based EC supercapacitor materials in Table S1 of SI. The comparison revealed a higher coloration efficiency and capacitance for the Fe-3TPY system with a comparable energy and power density to previous reported materials. Finally, we have demonstrated a proof-of-concept LED bulb illumination experiment using our lab-built ITO/Fe-3TPY-/LiClO_4_/ITO-based ECESD (Figure 6c), with a diameter of approximately 1.5 × 1.5 cm^2^. The prepared three ECESD in series connection effectively illuminated 10-blue LED bulbs (∼2.8 V each) panel connected by parallel connection, as shown in Figure 6d, once the devices were fully charged. The emission brightness of the LED panel gradually reduced as the devices regained their color from pale yellowish (charge state) to pristine pink (discharge state) over time, as shown in Figure 6d–g. Therefore, the EC color-indexed hybrid energy storage device has the potential to be used as a visually monitored energy storage management system. As the reference, the air-referenced transmittance of the devices is also provided in Figure S7, Supporting Information.

## Conclusions

In summary, the as-synthesized nonconjugated Fe-3TPY CONASH was applied in a hybrid ECESD by assembling NiHCF as CE and Li^+^ gel electrolyte. The electrochemical analysis revealed the predominant pseudocapacitive behavior (∼55% at 200 mV s^–1^ scan rate) of the ECESD during the redox transition. The fabricated ECESD displayed remarkable EC properties from pristine purple to pale yellowish, sustainable optical contrast (57.4% at 556 nm), rapid coloration and bleaching times (1.28 and 1.69 s), and a very high coloration efficiency of 619 cm^2^ C^–^^1^ with very less energy consumption (3.6 mJ/cm^2^) compared to the commercial display devices. Importantly, the ECESD revealed an excellent 50,000 EC cyclic durability and a very high optical memory (only ∼33% color retention after 36 h), indicating the robustness and energy efficiency of the EC device. Additionally, the ECESD also disclosed a high volumetric capacitance of 248.1 F/cm^3^ at 1 A/cm^3^ along with a high energy and power density of 29.37 mW h/cm^3^ and 7.5 W/cm^3^, respectively. The ECESD also demonstrates 40,000 continuous GCD performance and represents an attractive example for color-indicative energy storage devices for practical applicability. This study offers the energy efficiency of ECESDs in different aspects and the device’s robust performance for a prolonged period, with prospective applications in next-generation energy storage, enriching EC energy storage technology.

## Supplementary Material



## Notes

Details of the synthesis and characterizations of the CONASH; fabrication of the hybrid ECESD; calculations; thickness measurements; CV; UV; and GCD studies (PDF)
