# Supplementary material for: Coordination Nanosheet-Based Electrochromic Supercapacitor with High Energy Storage, Switching Durability, and Long Optical Memory Properties
Source: ACS Appl Mater Interfaces. 2025 Oct 29;17(45):62499–509. doi: 10.1021/acsami.5c13795 (PMC12616605; doi:10.1021/acsami.5c13795)
Supplement: Supplementary file 1 [file am5c13795_si_001.pdf]

Supporting Information for

Coordination Nanosheet-Based Electrochromic Supercapacitor with High Energy Storage, Switching Durability, and Long Optical Memory Properties

Susmita Roy,[a,b] Sayan Halder,[a] Sarda Sharma,[c] Karumbaiah N. Chappanda,[d] Chanchal Chakraborty,*[,a,e] Masayoshi Higuchi*[,b]

[a] Department of Chemistry, Birla Institute of Technology & Science (BITS) Pilani, Hyderabad Campus Jawaharnagar, Samirpet, Hyderabad, Telangana 500078, India

[b] Electronic Functional Macromolecules Group, National Institute for Materials Science (NIMS), 1-1 Namiki, Tsukuba, 305-0044 Japan

[c] Department of Electronics and Communication Engineering, Amrita School of Engineering, Bengaluru, Amrita Vishwa Vidyapeetham, 560035, India

[d] Sensors and Nano Electronics (SANE) Lab, School of Applied Engineering and Technology, Southern Illinois University Carbondale, IL 62901, USA

[e] Materials Center for Sustainable Energy & Environment (McSEE), Birla Institute of Technology and Science, Hyderabad Campus, Hyderabad 500078, India

Corresponding Authors:
Dr. Masayoshi Higuchi (Email: HIGUCHI.Masayoshi@nims.go.jp)
Dr. Chanchal Chakraborty (Email: chanchal@hyderabad.bits-pilani.ac.in)

Table of Contents

**Sr. No.**	**Content**	**Page**
1.	Synthesis of Fe-3TPY CONASH	S3
2.	Important calculations to determine index for electrochromic materials and for energy storage	S3
3.	Film thickness, UV-vis, and CV spectra of NiHCF-coated film	S5
4.	GCD measurement ECESD	S6
5.	The post-electrochemical morphology analysis of Fe-3TPY	S6
6.	EC performance of a relatively larger EC device	S7
7.	Energy storage study of polyFe ECESD without NiHCF layer	S7
8.	Energy storage study of Fe-3TPY ECESD without NiHCF layer	S8
9.	EC performance of the Fe-3TPY ECESD without background subtraction	S9

1. Synthesis of Fe-3TPY CONASH

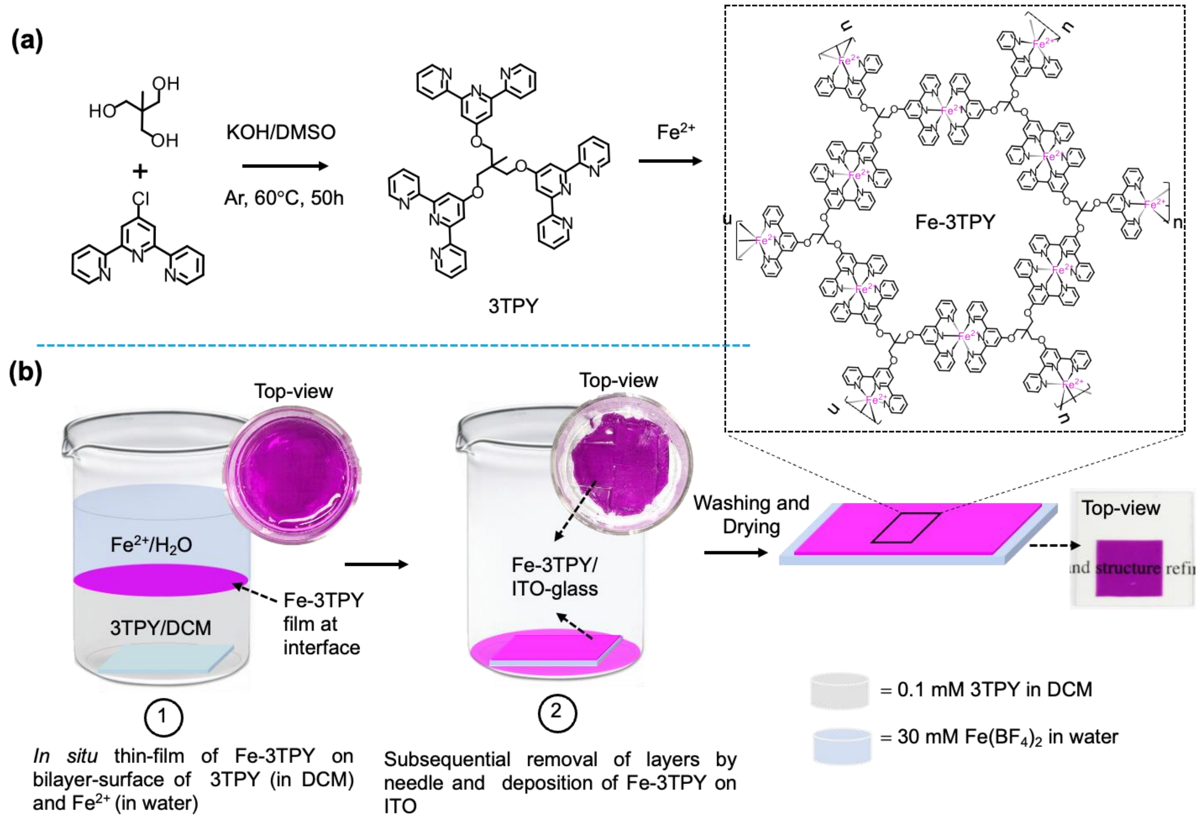

Scheme S1. (a) Chemical structure of synthesized Fe-3TPY CONASH by the coordination polymerization of 3TPY ligand and $Fe(BF_4)_2$. (b) Synthetic step for in situ film formation of Fe-3TPY CONASH from the constituent ligand and metal precursor at aqueous-organic bi-layer interface.

2. Important calculations to determine the index for electrochromic materials and for energy storage

The optical contrast (% ΔT) of the electrochromic device can be determined by,

$$\%\ \Delta T = \%\ T_{@\ +0.01\ V} - T_{@\ +1.0\ V} \ldots\ldots\ldots\ldots\ldots\ldots\ldots\ldots\ldots\ldots\ldots\ldots\ldots \quad (1)$$

The coloration efficiency (CE) can be calculated by using the eq. (2),

$$\eta = \frac{\Delta \mathrm{OD}}{Q_{\mathrm{d}}} = \log\frac{T_{\mathrm{b}}}{T_{\mathrm{c}}} \,/\, Q_c \ldots\ldots\ldots\ldots\ldots\ldots\ldots\ldots\ldots\ldots\ldots\ldots\ldots \quad (2)$$

Where ΔOD and Q_c are the change in optical density and amount of charge injected or ejected per unit area, T_b and T_c are the transmittance at bleached and colored states.

Calculation for coloration energy consumption

The coloration energy consumption of an electrochromic device can be calculated from the following equation. (3),

$$W/A = \frac{(V\int I(t)dt)}{A} = \frac{\int P(t)dt}{A} = V.Q/A = V.Q_d \quad (3)$$

Here, W represents coloration energy consumption (mJ), A is the effective surface area (cm^2), V is the voltage of the device (V), I denotes the generated current during EC color change (mA), t represents coloration time (s), P denotes the coloration power of the device in mW and Q signifies the total charge for coloration. The energy storage property was calculated using the galvanostatic charge-discharge (GCD) of the ECESD.

Calculations for the Electrochromic energy storage study

The volumetric capacitance (C_v in F/cm^3), the energy density (E in Wh/cm^3), and the power density (P in W/cm^3) can be evaluated from the GCD curves using Equations 4-6, respectively:

$$C_v = \frac{i}{v} \times \frac{\Delta t}{\Delta V} \quad (4)$$

$$E = \frac{1}{2} \times C_v \times \frac{(\Delta V)^2}{3600} \quad (5)$$

$$P = \frac{E}{\Delta t} \times 3600 \quad (6)$$

Here, i, Δt, ΔV, and v denote the input current and discharge time obtained from the galvanostatic charge-discharge (GCD) plot, potential window, and film volume, respectively.

3. Film thickness, UV-vis and CV spectra of NiHCF-coated film

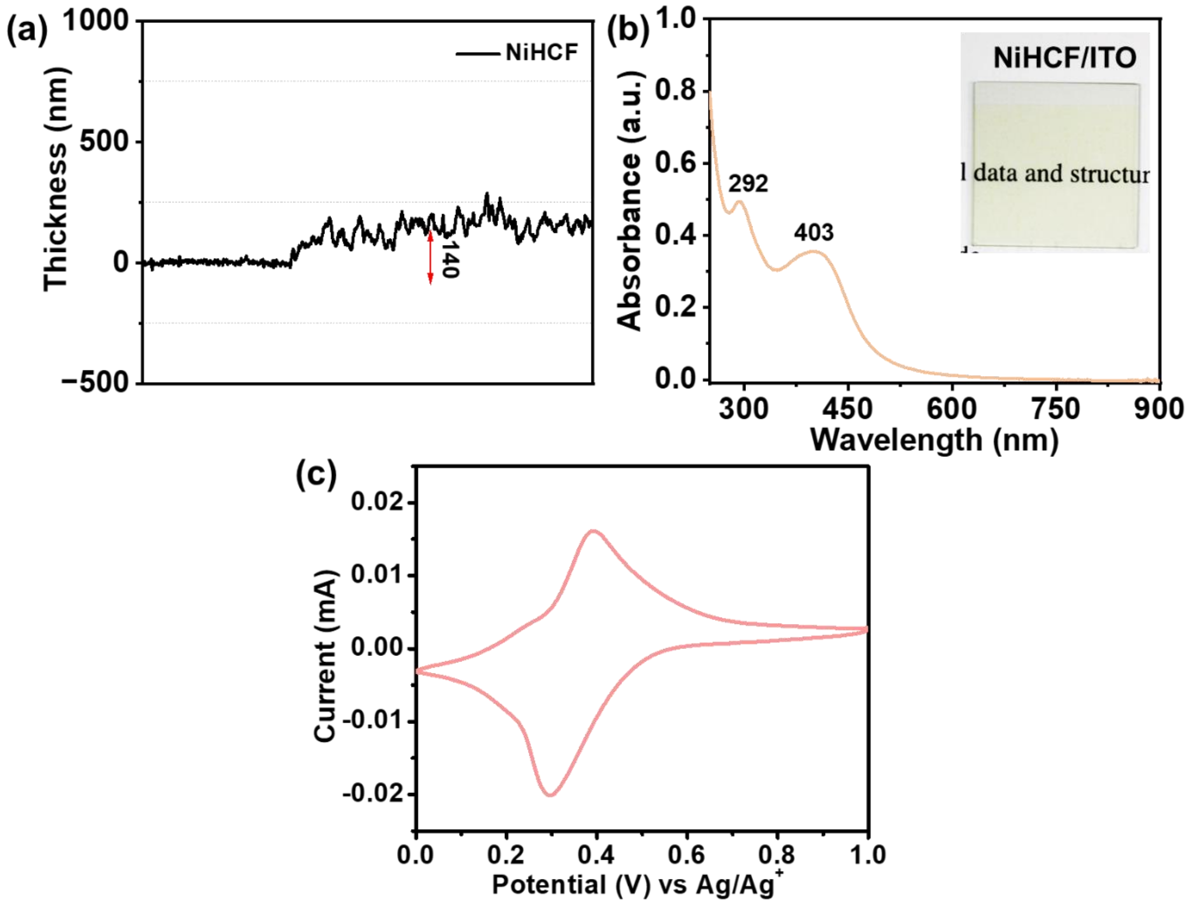

Figure S1. (a) The measured thickness of the NiHCF film by the line profilometer. (b) The absorbance spectra of the NiHCF film. A picture of transparent NiHCF-coated ITO-glass is given in the inset. (c) The CV study of NiHCF film in a three-electrode system using Ag/AgCl as the reference electrode, Pt wire as the counter electrode, and 0.1 M $LiClO_4$-acetonitrile electrolyte at a scan rate of 50 mV/s.

4. GCD measurement ECESD

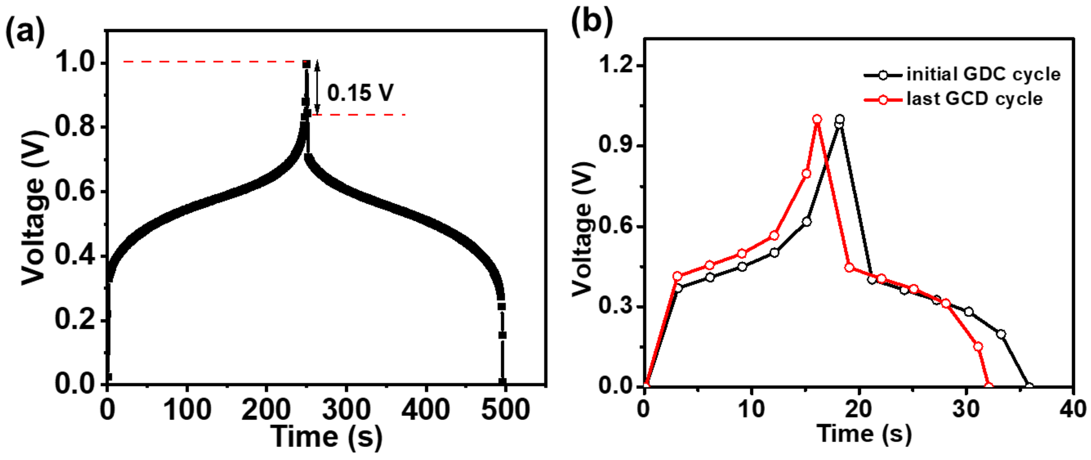

Figure S2. (a) The IR drop measurement of the ECESD at 1 A/cm^3 current density. (b) The initial and final GCD cycle for the ECESD.

5. The post-electrochemical morphology analysis of Fe-3TPY

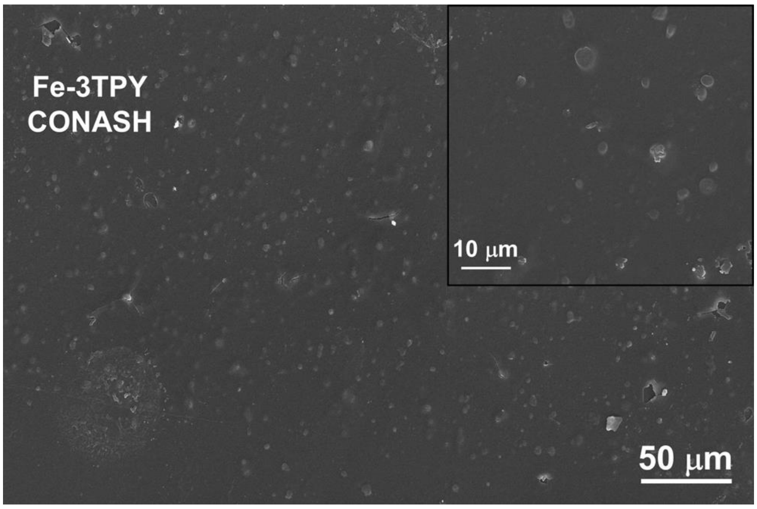

Figure S3. FESEM of Fe-3TPY film after prolonged EC performance.

6. EC performance of a relatively larger EC device of the Fe-3TPY film

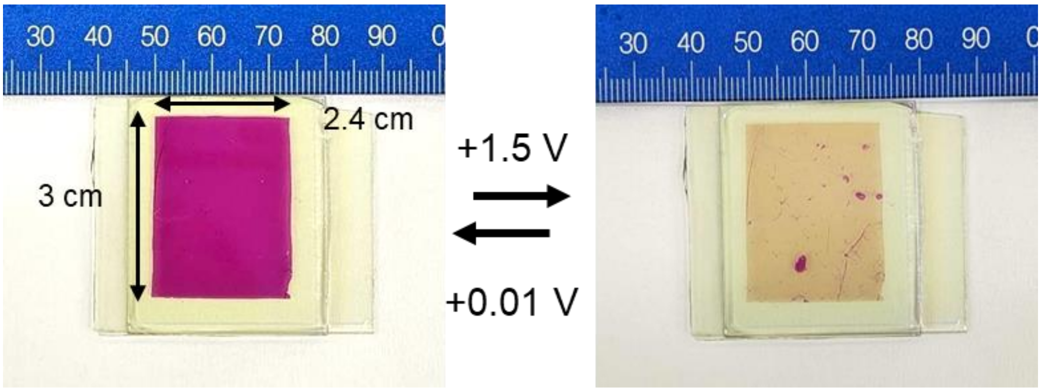

Figure S4. EC switching of a larger device (Fe-3TPY/$LiClO_4$-PC-PMMA-MeCN/NiHCF)

7. Energy storage study of conjugated polyFe ECESD with NiHCF layer

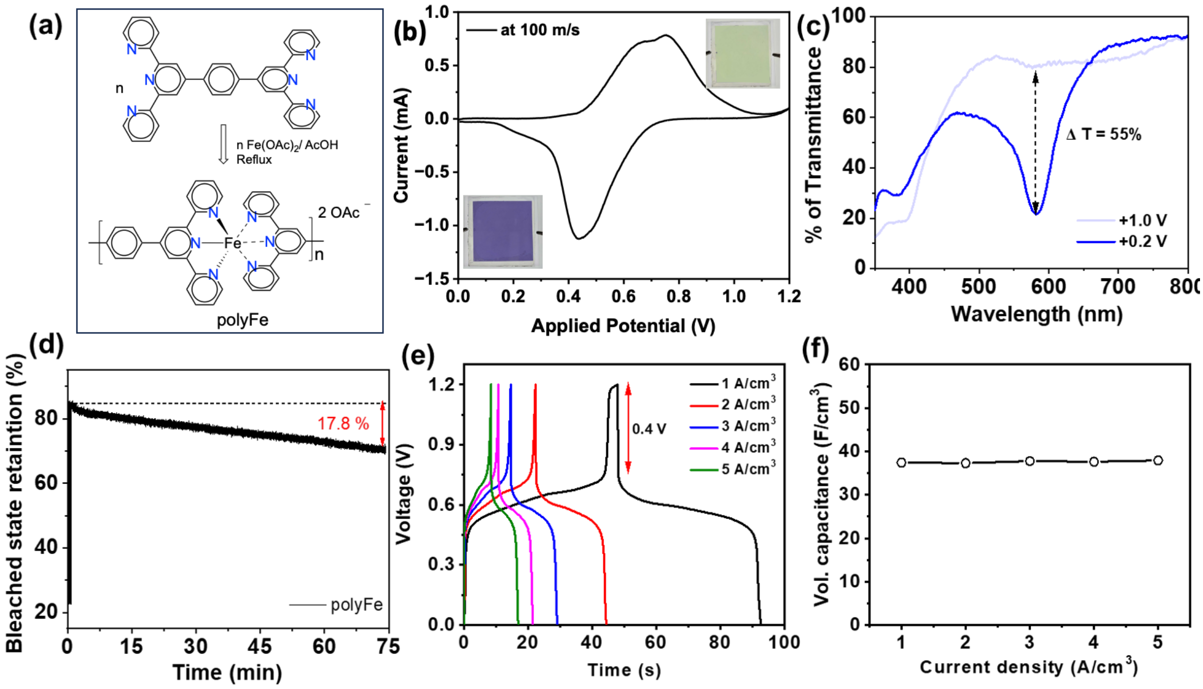

Figure S5. (a) Synthesis of polyFe. (b) The CV study of ITO/polyFe/$LiClO_4$/NiHCF/ITO-based ECESD. (c) GCD profiles and (d) volumetric capacitance plot at various current densities ranging from 1 to 5 A/cm³. (e) The electrochromic transmittance changes at different applied voltages, and (f) transmittance decay plots at open-circuit conditions for ITO/polyFe/$LiClO_4$/ITO-based ECESD.

8. Energy storage study of Fe-3TPY ECESD without NiHCF layer

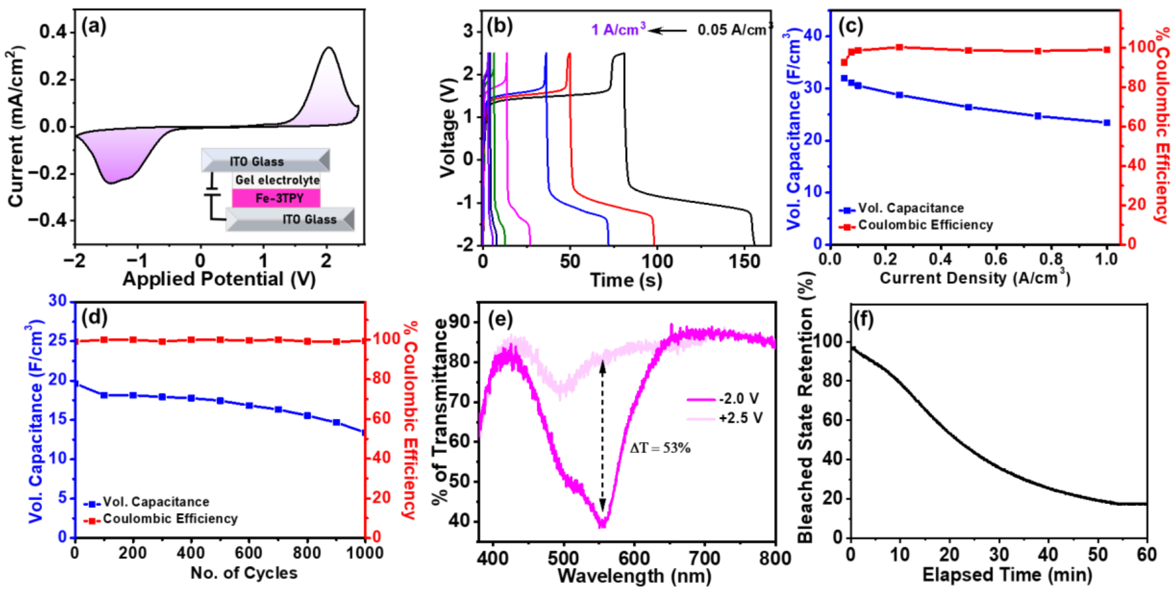

Figure S6. (a) The CV study of ITO/Fe-3TPY/$LiClO_4$/ITO-based ECESD. (b) GCD profiles and (c) volumetric capacitance plot at various current densities ranging from 0.05 to 1 A/cm³. (d) GCD stability plot ITO/Fe-3TPY/$LiClO_4$/ITO-based ECESD. (e) The electrochromic transmittance changes at different applied voltages, (f) transmittance decay plots at open-circuit conditions for ITO/Fe-3TPY/$LiClO_4$/ITO-based ECESD.

9. EC performance of the Fe-3TPY ECESD without background subtraction

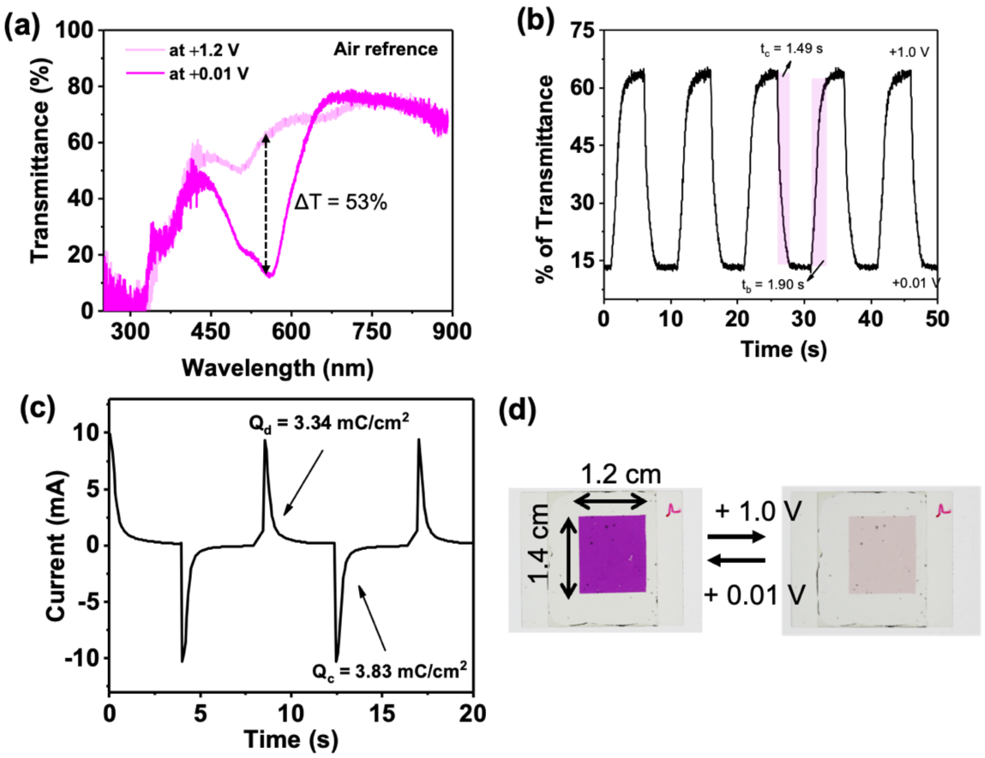

Figure S7. (a) The transmittance spectral change of ITO/Fe-3TPY/$LiClO_4$/ITO-based ECESD was measured without background subtraction. (b) The EC response time determination of the ECESD is based on a 95% change in transmittance. (c) Chronoamperometry charge-discharge plot during the electrochromism of the ECESD. (d) Photograph of the color change for the fabricated ECESD (film size: 1.4 × 1.2 cm) at different voltages.

Table S1: Comparison of electrochromic and energy storage properties with previously reported MSPs.

MSPs	Color change in EC with *ΔT%*	t_c/t_b (s/s)	CE (η) (cm^2 C^{-1})	Capacitance	Energy density	Power density	Ref.
PolyFe (1D MSP)	Purple to light green	0.35/0.35	315	39 ± 2 F/cm^3 at 1 A/cm^3	10.6 mWh/cm^3	7 W /cm^3	[1,2]
Fe-TpyTzTpy (1D MSP)	Purple taupe to yellow	0.32/2.7	324	21 F/cm^3 at 1 A/cm^3	13 mWh/cm^3	5 W/cm^3	[3]
Fe-TpyNTpy (1D MSP)	Purple to dirty white 48.5% at 570 nm	3.2/2.2	340.5	73 ± 2 F/cm^3 at 1 A/cm^3	61 mWh/cm^3	8.5 W/cm^3	[4]
Fe-BTZ (1D MSP)	Matte purple to dirty yellow 65.2% at 567 nm	1.1/1.9	1077	70.1 ± 4 F/cm^3 at 1 A/cm^3	21.3 mWh/cm^3	8 W/cm^3	[5]
polyFe-2D (2D MSP)	red to colorless 56%	2.9/3.3	230	82 ± 5 F/cm^3 at 1 A/cm^3	13.8 mWh/cm^3	15.1 W/cm^3	[6,7]
2Phen-TPA (2D MSP)	orange to deep green 54% at 625 nm	4.8/5.5	134	8.12 mF/cm^2 at 0.1 mA/cm^2	10.37 μWh/cm^2	338.8 μW/cm^2	[8]
MSP-Fe (2D MSP)	purple to yellow 71%	0.5/1.5	1103.9	12.01 mAh/g at 0.2 A/g	0.93 Wh/kg	285 W/kg	[9]
TTP-Fe (3D MSP)	purple to yellow 63%	3.6/2	486	29.12 F/g at 0.4 A/g	-	-	[10]
poly-Fe-L2 (3D MSP)	purple to yellow	2.3/1.6	-	544.6 F/g at 0.25 A/g	75.5 mWh/cm^3	0.154 kW/cm^3	[11]
**Fe-3TPY (2D MSP)**	**Pink to pale yellowish 57.4% at 556 nm**	**1.28/1.69**	**619**	**248.1 F/cm^3 at 1 A/cm^3**	**29.37 mWh/cm^3**	**7.5 W/cm^3**	**This work**

References:

1. Mondal, S.; Yoshida, T.; Maji, S.; Ariga, K.; Higuchi, M. Transparent Supercapacitor Display with Redox-Active Metallo-Supramolecular Polymer Films, *ACS Appl. Mater. Interfaces* **2020**, *12*, 16342–16349.
2. Mondal, S.; Roy, S.; Fujii, S.; Higuchi, M. Highly Durable Electrochromic Devices for More than 100,000 Cycles with Fe(II)-Based Metallo-Supramolecular Polymer by Optimization of the Device Conditions, *ACS Appl. Electron. Mater.* **2023**, *5*, 6677–6685.
3. Halder, S.; Chakraborty, C. Fe(II)-based dual function metallo-supramolecular polymer with thiazolothiazole spacer for high-performance electrochromic supercapattery, *Sol. Energy Mater. Sol. Cells* **2023**, *254*, 112288.
4. Halder, S.; Garg, S.; Chakraborty, C. Introducing non-conjugated ionic spacer in metallo-supramolecular polymer: Generation of nanofibers for high-performance electrochromic supercapacitor, *Chem. Eng. J.* **2023**, *470*, 144361.
5. Halder, S.; Chakraborty, C. Ligand-engineered Fe(II)-metallo-supramolecular polymer with benzothiadiazole: Boosting electrochromic energy storage efficiency, *Chem. Eng. J.* **2024**, *498*, 155382.
6. Mondal, S.; Ninomiya, Y.; Yoshida, T.; Mori, T.; Bera, M.K.; Ariga, K.; Higuchi, M. Dual-Branched Dense Hexagonal Fe(II)-Based Coordination Nanosheets with Red-to-Colorless Electrochromism and Durable Device Fabrication, *ACS Appl. Mater. Interfaces* **2020**, *12*, 31896–31903.
7. Mondal, S.; Ninomiya, Y.; Higuchi, M. Durable Supercapattery Film with Dual-Branched Dense Hexagonal Fe(II)-Based Coordination Nanosheets for Flexible Power Sources, *ACS Appl. Energy Mater.* **2020**, *3*, 10653–10659.
8. Cong, B.; Xie, Y.; Wu, Y.; Zhou, H.; Chen, C.; Zhao, X.; Chao, D. Metal-organic coordination polymer bearing dual-redox centra enables high-performance electrochromic supercapacitor, *Chem. Eng. J.* **2023**, *474*, 145528.
9. Liu, S.; Wei, C.; Wang, H.; Yang, W.; Zhang, J.; Wang, Z. Zhao, W.; Lee, P.S.; Cai, G. Processable nanoarchitectonics of two-dimensional metallo-supramolecular polymer for electrochromic energy storage devices with high coloration efficiency and stability, *Nano Energy* **2023**, *110*, 108337.
10. Chen, H.; Xing, J.; Wang, W.; Li, X.; Shu, M.; Gao, P.; Pan, Y.; Liu, J. Electrochromic and energy storage properties of novel terpyridine-Fe(II) coordination polymers:

Improving performance by molecular engineering of nonconjugated linkers from linear to three-arm star configuration, *Sol. Energy Mater. Sol. Cells* **2022**, *248*, 111967.

11. Mukkatt, I.; Mohanachandran, A.P.; Nirmala, A.; Patra, D.; Sukumaran, P.A.; Pillai, R.S.; Rakhi, R.B.; Shankar, S.; Ajayaghosh, A. Tunable Capacitive Behavior in Metallopolymer-based Electrochromic Thin Film Supercapacitors, *ACS Appl. Mater. Interfaces* **2022**, *14*, 31900–31910.
